# Prevalence and Factors Associated With Elderly Abuse and Health Problems Among Elderly People of Kathmandu Valley, Nepal

**DOI:** 10.1155/jare/9932315

**Published:** 2026-01-05

**Authors:** Lochana Shrestha, Naresh Manandhar, Leela Paudel, Ganesh Bhandari, Ayushma Poudel

**Affiliations:** ^1^ Department of Community Medicine, College of Medicine, Nepalese Army Institute of Health Sciences (NAIHS), Sanobharang, Kathmandu, Bagmati, Nepal, medcol.mw; ^2^ Health Office, Ministry of Social and Development, Kanchanpur, Sudurpashchim, Nepal

**Keywords:** elder abuse, health problem, prevalence

## Abstract

**Background:**

The global population of older adults is expanding in developing nations. According to the WHO, elder abuse is defined as “a single or repeated act, or lack of appropriate action, occurring within any relationship where there is an expectation of trust, which causes harm or distress to an older person.” Such abuse is a global concern. This study aimed to find the prevalence, contributing factors, and impacts to inform effective prevention and intervention strategies.

**Methods:**

A community‐based cross‐sectional study was conducted via face‐to‐face interviews with elderly individuals aged 60 years and above. The study was conducted among participants from the Kathmandu Valley, Nepal. The sample size was 385 with a prevalence of 50.3%. A multistage sampling technique was employed to select the sample. The first household was chosen randomly, and subsequent households were selected using the calculated sampling interval.

**Results:**

Psychological/emotional abuse was the most common (18.7%), followed by financial abuse, physical abuse, and neglect with sexual abuse reported rarely. The most frequently reported health problem was hypertension (21.4%) followed by osteoarthritis/osteoporosis and diabetes. Gender shows a significant association with emotional abuse (*p* = 0.02), where females (24.4%) experience higher rates compared to males (14.8%). Physical abuse is more common among females (16.7%) than males (10.0%), though the difference is marginally insignificant (*p* = 0.06).

**Conclusions:**

Elderly abuse has been increasingly recognized as a serious global issue, warranting urgent attention from healthcare systems and national authorities.

## 1. Introduction

The global population of older adults is expanding more rapidly than any other age group, especially in developing nations where the pace of population aging is significantly faster than it was in developed countries historically [1]. The World Health Organization (WHO) defines health as “a state of complete physical, mental, and social wellbeing, not merely the absence of disease or infirmity.” Aging brings physiological changes, such as deterioration in vision, hearing, memory, and motor coordination, due to the degeneration of bodily systems and the weakening of the immune system. This process affects vital organs, which gradually lose their function as people age. According to the WHO, elder abuse is defined as “a single or repeated act, or lack of appropriate action, occurring within any relationship where there is an expectation of trust, which causes harm or distress to an older person” [2]. Such abuse, which may be committed by family members, friends, caregivers, or neighbors, is a global concern. It can occur through acts of commission or omission and includes physical, sexual, emotional, and financial abuse, as well as neglect, abandonment, and self‐neglect. Elderly abuse has been increasingly recognized as a serious global issue, calling for urgent attention from healthcare systems and national authorities [3].

Elder abuse often has profound negative consequences, including physical injuries, disability, mental health issues, substance abuse, and chronic diseases. The consequences of elder abuse extend to increased healthcare needs and impact social services and judicial systems [4]. Approximately 1 in 6 people aged 60 and above has experienced some form of abuse in community settings within developing countries. A 2017 review of 52 studies across 28 countries, including 12 low‐ and middle‐income countries, estimated a 15.7% prevalence of elder abuse [5]. In Nepal in 2021, a study found that 47.7% of older adults reported experiencing at least one form of abuse in the past 6 months, with neglect being the most common, followed by disrespect, verbal abuse, economic abuse, physical abuse, and emotional abuse [6]. Factors associated with elder abuse include poor physical and mental health, mental disorders, substance abuse, and financial dependency of abusers on the elderly and low financial status or limited education of the older adult. Elderly women, especially those who are widowed, are at higher risk of abuse. Other factors, as identified in studies, include age, gender, marital status, educational level, family dynamics, social support, solitude, and dependency on others for daily activities.

Despite the rising prevalence, elder abuse remains understudied in Nepal from a community perspective. This study examines the physical, mental, and social factors associated with elder abuse, providing insights for policy and program development. Although global studies link health issues to increased vulnerability, research in Nepal is limited. Comparisons with neighboring countries such as India highlight varying abuse rates and health concerns. In addition to India, several important studies on elder abuse have emerged from Southeast Asia. Notably, the Malaysian Elder Mistreatment Project (MAESTRO) examined prevalence and determinants of elder abuse in Kuala Lumpur (Choo WY et al., BMJ Open 2016; 6:e010284) [7]. Subsequent analyses from this cohort (BMC Geriatrics 2018; 18:195; Front Public Health 2021) reported emotional and financial abuse as the most common types, emphasizing the need for regionally sensitive measurement tools and interventions [8]. A study conducted in a rural community in Malaysia found that at least one‐third of the elderly expressed that they had not heard of financial abuse before. A few elderly individuals mentioned that they had heard it somewhere and were aware of it. However, many elderly individuals were able to relate to older adults being cheated of their money or property. Participants expressed that acts of cheating and taking advantage of others for financial gains are frequent events that happen in society. More than half of the elderly expressed worries and concerns that financial abuse and exploitation can occur without their knowledge [9].

A study conducted by Bonnie RJ and Wallace RB revealed that elderly individuals are also financially motivated and can involve pressure for gifting money or assets, shaming to move into care, or unmet promises to take care of the older person if they transfer assets to the child’s name [10].

A study conducted by Kaspiew and Carson in Australia found that elder abuse is often intergenerational, such as where a parent is abused by their adult child [11]. A study conducted by Qu et al. in Melbourne, Australia, revealed that one in six (15%) elderly Australians will experience abuse in the community, and the Royal Commission into Aged Care Quality and Safety estimates that 39.2% of elderly Australians living in nursing homes will experience elder abuse [12].

A study conducted by Sooryanarayana in Kuala Pilah District, Negeri Sembilan state of Malaysia, found that the prevalence of overall abuse was 4.5% in the past 12 months. Psychological abuse was most common, followed by financial, physical, neglect, and sexual abuse. Two or more occurrences of abusive acts were common, whereas clustering of various types of abuse was experienced by one‐third of abused elders [13].

These studies highlight the scarcity of research from Nepal and provide a valuable regional context for understanding elder abuse patterns across South and Southeast Asia. This study aims to assess prevalence, contributing factors, and impacts to inform effective prevention and intervention strategies.

## 2. Methods

A quantitative method was used to collect data through the administration of a structured questionnaire. A community‐based cross‐sectional study was conducted. This study employed a cross‐sectional design, carried out via face‐to‐face interviews with elderly individuals aged 60 years and above. The study population consisted of elderly individuals aged 60 years and above who had been residing at their current location for at least 6 months. The sampling unit included elderly individuals aged 60 years and above.

The sample size was calculated to represent the selected province. To derive the sample size, the prevalence of 50.3% was taken from a study conducted by Chalise et al. The sample size was 385.

A multistage sampling technique was used to select participants from Bagmati Province, Nepal, to estimate elderly abuse prevalence. Formal permissions were obtained from relevant authorities. Enumerators explained the study in simple language, obtained written informed consent, and provided an information sheet detailing objectives, data collection methods, potential benefits, and risks. For illiterate participants, consent was given via thumbprint in the presence of family members.

Face‐to‐face interviews were conducted privately using structured questionnaires. Data collection was supervised by the principal and coinvestigators, checked for completeness, and manually edited the same day. Data were analyzed using univariate and bivariate methods, including the chi‐square test for associations. This study was conceptually guided by the WHO Ecological Model of Elder Abuse, which explains elder mistreatment as resulting from the interaction of factors at individual, relational, community, and societal levels. This framework informed the study’s focus on demographic, behavioral, and health‐related determinants of abuse.

Ethical approval was obtained from the Institutional Review Committee of NAIHS (Reg. No. 1090, July 26, 2024), and findings will be used for research purposes only. The questionnaire was designed based on the WHO Elder Abuse Prevalence Study Instrument (2016) and the WHO Decade of Healthy Aging guidelines for elder abuse studies [14] and the Elder Abuse Assessment Tool Kit: Breaking the Silence: Giving a Voice Back to Seniors produced by the Durham Elder Abuse Network [15]. The five major categories—physical, psychological, sexual, financial, and neglect—were operationalized following WHO definitions but were culturally adapted for use in Nepal. The adaptation process involved translation, expert panel review, and pretesting to ensure cultural relevance and clarity. Although WHO measures were referenced for continuity, certain questions were simplified to enhance comprehension among elderly participants.

## 3. Results

The findings related to sociodemographic characteristics of elderly individuals are shown in Table 1. The study includes participants from three districts of Kathmandu Valley—Bhaktapur (35.1%), Kathmandu (34.8%), and Lalitpur (30.1%). Among the municipalities, the highest representations of participants were from Changunarayan (13.2%) and Suryabinayak (12.2%). The respondents’ ages range from 60 to 95 years, with an average of 69 years. Males make up 59.5% of the sample. Ethnically, Brahmin/Chhetri (51.7%) and Janajati (47%) groups predominate. Most participants are Hindu (89.9%). The predominance of Hindu and Brahmin/Chhetri participants reflects the demographic composition of urban Kathmandu Valley, where these groups form the majority. However, this profile may represent relatively advantaged socioeconomic groups compared to rural or marginalized populations, and thus, the findings may not fully represent Nepal’s broader elderly population.

**Table 1:** Sociodemographic characteristics of participants [2, 3].

Variable	No. of respondents (*n*)	Percentage (%)
Name of district		
Bhaktapur	135	35.1
Kathmandu	134	34.8
Lalitpur	116	30.1
Municipality		
Bhaktapur	38	9.9
Changunarayan	51	13.2
Godawari	41	10.6
Gokarneshwor	45	11.7
Kirtipur	45	11.7
Lalitpur	35	9.1
Mahalaxmi	39	10.1
Nagarjun	44	11.4
Suryabinayak	47	12.2
Age (in years)		
≤ 68	202	52.5
> 68	183	47.5
Mean = 69, Md = 68, Sd = 7.07, Min = 60, Max = 95		
Sex		
Male	229	59.5
Female	156	40.5
Ethnicity		
Brahmin/Chhetri	199	51.7
Janajati	181	47
Dalit	5	1.3
Religion		
Hinduism	346	89.9
Buddhism	16	4.2
Christianity	21	5.5
Kirat	2	0.5
Marital status		
Married	289	75.1
Separated	9	2.3
Unmarried	3	0.8
Widow	84	21.8
Educational status		
Illiterate	138	35.8
Informal	86	22.3
Basic	70	18.2
Secondary	51	13.2
Graduate	28	7.3
Postgraduate or higher	12	3.1
Employment status		
Currently employed	43	11.2
Unemployed	342	88.8
Live with		
Alone	22	5.7
With spouse	75	19.5
With spouse and children	187	48.6
With children only	88	22.9
Relatives/others	13	3.4
Number of children with you		
0	17	6.2
1	170	61.8
2	71	25.8
3	9	3.3
4	8	2.9
Mean = 1.35, Md = 1, Sd = 0.77, Min = 0, Max = 4		
Number of children not with you		
0	95	34.5
1	81	29.5
2	52	18.9
3	28	10.2
4	11	4.0
5	6	2.2
6	2	0.7
Mean = 1.29, Md = 1, Sd = 1.32, Min = 0, Max = 6		
Total number of children		
1	45	16.4
2	108	39.3
3	62	22.5
4	35	12.7
5	14	5.1
6	8	2.9
7	3	1.1
Mean = 2.64, Md = 2, Sd = 1.30, Min = 1, Max = 7		
Personal savings for future		
Yes	212	55.1
No	173	44.9

Regarding marital status, 75.1% are married, whereas 21.8% are widowed. Educational attainment varies, with 35.8% being illiterate and 7.3% having graduate‐level education. Most respondents (88.8%) are unemployed. In terms of living arrangements, 48.6% live with both spouse and children, whereas 5.7% live alone. The average number of children is 2.64, with 1.35 children living with them and 1.29 children away.

Financially, 55.1% have personal savings, whereas 44.9% do not, highlighting a divide in financial security among the elderly respondents.

The findings related to behavioral factors of elderly individuals are shown in Table 2. Among the 385 participants, 139 (36.1%) reported consuming alcohol, whereas 246 (63.9%) did not. Among 139 who consume alcohol, 27.3% drink daily, 18.7% drink weekly, 8.6% drink monthly, and 45.4% drink occasionally. Regarding smoking habits, 112 (29.1%) participants reported smoking, whereas 273 (70.9%) did not. Among the 112 smokers, 85.7% smoke daily, 5.4% smoke weekly, and 8.9% smoke occasionally.

**Table 2:** Behavioral factors of study participants [2, 3].

Variable	Frequency (*n*)	Percentage (%)
Consume alcohol		
Yes	139	36.1
No	246	63.9
Frequency of alcohol intake (*n* = 139)		
Daily	38	27.3
Weekly	26	18.7
Monthly	12	8.6
Occasionally	63	45.4
Smoking		
Yes	112	70.9
No	273	29.1
Frequency of smoking (*n* = 112)		
Daily	96	85.7
Weekly	6	5.4
Occasionally	10	8.9

The findings related to elderly abuse are shown in Table 3. The analysis revealed several forms of abuse—physical, emotional, sexual, financial/material, and cases of neglect—highlighting the vulnerability of the elderly population. Of 385 participants, 49 (12.7%) reported experiencing physical abuse. Among the 49 physical abuses, 55.1% were beaten, 83.7% were pushed, and 55.1% had their arms gripped until painful. These results suggest that physical abuse, although less prevalent, can be severe when it occurs. Emotional or psychological abuse was reported by 72 (18.7%) participants. Among 72 emotional abuses, 79.2% were insulted, 38.9% were threatened, and 20.8% were restricted from visiting neighbors. Additionally, 36.1% were afraid of someone in their family, whereas 9.7% were threatened with being sent to an old age home, and 5.6% faced death threats. Emotional abuse was the most common form of abuse, reflecting significant psychological distress among the elderly. Sexual abuse was reported by only three (0.8%) participants. Among these cases, 66.7% reported being touched inappropriately, whereas 33.3% faced unwelcome sexual advances. Although rare, sexual abuse represents a critical concern. Financial abuse was reported by 65 (16.9%) participants. Among 65 financial abuses, 90.8% had their money borrowed without repayment, 27.7% experienced unauthorized asset transfers, and 47.7% had their money used for others’ benefit. Financial exploitation poses a significant risk to the elderly’s financial security. Neglect or abandonment was reported by 40 (10.4%) participants. Of the 40 individuals identified as neglected, 72.5% felt unattended during their time of need and 72.5% felt their needs were unfulfilled. However, 85% reported that treatment was arranged when they were sick, whereas 57.5% felt well attended during illness.

**Table 3:** Physical, emotional, sexual, and financial abuse of elderly individuals [2].

Variable	Frequency (*n*)	Percentage
*Physical abuse*		
Physical abuse (*n* = 385)		
Yes	49	12.7
No	336	87.3
Beaten (*n* = 49)		
Yes	27	55.1
No	22	44.9
Pushed (*n* = 49)		
Yes	41	83.7
No	8	16.3
Gripped arm (*n* = 49)		
Yes	27	55.1
No	22	44.9

*Emotional abuse*		
Emotional/psychological abuse (*n* = 385)		
Yes	72	18.7
No	313	81.3
Insulted (*n* = 72)		
Yes	57	79.2
No	15	20.8
Threatened (*n* = 72)		
Yes	28	38.9
No	44	61.1
Left to unknown place (*n* = 72)		
Yes	3	4.2
No	69	95.8
Limit to visit neighbor (*n* = 72)		
Yes	15	20.8
No	57	79.2
Afraid of anyone (*n* = 72)		
Yes	26	36.1
No	46	63.9
Threatened to send to old age home (*n* = 72)		
Yes	7	9.7
No	65	90.3
Threatened to kill (*n* = 72)		
Yes	4	5.6
No	68	94.4

*Sexual abuse*		
Sexual abuse (*n* = 385)		
Yes	3	0.8
No	382	99.2
Talked in sexual way (*n* = 3)		
Yes	1	33.3
No	2	66.7
Touched in sexual way (*n* = 3)		
Yes	2	66.7
No	1	33.3
Forced to engage in sexual act (*n* = 3)		
Yes	2	66.7
No	1	33.3
Tried to engage you in sexual experience (*n* = 3)		
No	3	100

*Financial abuse*		
Financial abuse (*n* = 385)		
Yes	65	16.9
No	320	83.1
Borrowed money (*n* = 65)		
Yes	59	90.8
No	6	9.2
Transferred assets (*n* = 65)		
Yes	18	27.7
No	47	72.3
Used money for benefit (*n* = 65)		
Yes	31	47.7
No	34	52.3
Refused to give account of money spent (*n* = 65)		
Yes	11	16.9
No	54	83.1

*Neglect or abandonment*		
Neglect or abandonment (*n* = 385)		
Yes	40	10.4
No	345	89.6
Feel attended in need (*n* = 40)		
Yes	11	27.5
No	29	72.5
Needs fulfilled (*n* = 40)		
Yes	11	27.5
No	29	72.5
Treatment arranged when sick (*n* = 40)		
Yes	34	85
No	6	15
Well attended during sickness (*n* = 40)		
Yes	23	57.5
No	17	42.5

The findings related to self‐reported health problems of elderly individuals are shown in Table 4. The analysis reveals a range of health issues commonly reported by the participants, underscoring the burden of chronic diseases and functional limitations in this demographic. The most frequently reported health problem was hypertension, affecting 21.4% of the participants. Hypertension is a prevalent condition among the elderly and a significant risk factor for other cardiovascular diseases. Joint pain and bone‐related issues, such as osteoarthritis or osteoporosis, were reported by 14.5% of the participants. This is a common concern among older adults due to age‐related degeneration of bones and joints. Diabetes was reported by 13.7% of the participants. Given the increasing prevalence of lifestyle‐related diseases, diabetes is a critical health issue among the elderly, requiring continuous management. Respiratory issues were noted by 8.1% of participants. Chronic respiratory conditions such as COPD and asthma are common in older adults, contributing to overall morbidity. About 4.7% of participants reported oral health issues, which can significantly impact nutrition and overall well‐being. Heart diseases, including myocardial infarction (MI) and stroke, were reported by 5.4% of participants, reflecting the cardiovascular risks prevalent in the elderly population. Hearing loss was reported by 5.7%, whereas 7.4% of participants experienced low eyesight. Cognitive decline or memory loss was reported by 4.5% of participants. This highlights the risk of dementia or related cognitive disorders in older adults. Urinary incontinence was reported by 3.4% of participants, whereas bowel incontinence affected 0.7% of participants. Falls were reported by 1.8% of the participants, indicating the risk of injury and frailty in this population. Less than 1%, that is, 0.7%, of participants reported functional disabilities, which can severely limit daily activities and independence.

**Table 4:** Self‐reported health problems of participants [2].

Variable (multiple response)	Frequency (*n*)	Percentage
Self‐reported health problems		
Functional disabilities	6	0.7
Diabetes	113	13.7
Hypertension	177	21.4
Memory loss (cognitive decline)	37	4.5
Oral health problems	39	4.7
Heart disease (MI/stroke)	45	5.4
Osteoarthritis or osteoporosis (knee pain)	120	14.5
Respiratory disease	67	8.1
Urinary incontinence	28	3.4
Falls	15	1.8
Bowel incontinence	6	0.7
Others	66	8.0
Hearing loss	47	5.7
Low eyesight	61	7.4

The findings related to the association between various forms of elderly abuse and several variables are shown in Table 5. This analysis explores the association between various sociodemographic factors and different forms of elderly abuse, including physical, emotional/psychological, financial/material abuse, and neglect or abandonment. Sexual abuse was excluded from this analysis due to its very low frequency (only three cases), which limits the statistical power and relevance of any conclusions drawn regarding this form of abuse. The results show no significant associations between the district and any form of abuse. However, it is observed that physical abuse is slightly more prevalent in Bhaktapur (15.6%) compared to Kathmandu (11.2%) and Lalitpur (11.2%). Emotional/psychological abuse is highest in Kathmandu (22.4%), followed by Lalitpur (19%) and Bhaktapur (14.8%). Financial/material abuse is notably higher in Lalitpur (23.3%) compared to the other districts, whereas neglect or abandonment is most prevalent in Kathmandu (14.2%). There is no significant difference in the prevalence of abuse based on age. However, it is noted that physical abuse is slightly higher among those aged 68 years or below (13.9%) compared to those aged above 68 (11.5%). Emotional/psychological abuse and financial/material abuse are more common in the older age group (> 68), with rates of 20.2% and 18.6%, respectively. Neglect is also more prevalent in the older age group (13.1%). Gender shows a significant association with emotional/psychological abuse (*p* = 0.02), where females (24.4%) experience higher rates compared to males (14.8%). Physical abuse is more common among females (16.7%) than males (10.0%), though the difference is marginally insignificant (*p* = 0.06). Financial/material abuse and neglect do not show significant gender differences, although females still report slightly higher rates. Ethnicity does not show significant associations with any form of abuse. However, Dalits report the highest rates of emotional/psychological abuse (40%) and neglect (20%), although the sample size is very small. Brahmin/Chhetris and Janajatis report similar rates of physical, emotional, and financial abuse. Religion does not show any significant associations with abuse. Hindu participants experience slightly lower rates of abuse across all forms compared to those of other religions (Buddhism, Christianity, and Kirat). Although marital status is not significantly associated with any form of abuse, emotional/psychological abuse is more common among those who are unmarried, widowed, or separated (25%) compared to those who are married (16.6%). Educational status shows significant associations with physical abuse (*p* = 0.01) and emotional/psychological abuse (*p* = 0.01). Illiterate individuals experience higher rates of physical abuse (18.8%) and emotional/psychological abuse (25.4%) compared to those who are literate. Employment status does not show significant associations with any form of abuse, though those who are unemployed report slightly higher rates of physical (13.5%) and emotional/psychological (19.9%) abuse compared to those who are employed. No significant associations are found between living arrangements and abuse. However, those living alone report lower rates of physical (4.5%) and emotional/psychological (4.5%) abuse compared to those living with others. The number of children not living with the elderly shows a significant association with physical abuse (*p* = 0.03). Those with fewer than two children not living with them report higher rates of physical abuse (16%) compared to those with two or more children (6.8%). Personal savings are not significantly associated with any form of abuse, though those without savings report slightly higher rates of physical and emotional abuse. Smoking is significantly associated with both physical abuse (*p* = 0.01) and neglect (*p* = 0.003). Smokers report lower rates of physical abuse (9.9%) compared to nonsmokers (19.6%), whereas nonsmokers experience higher rates of neglect (17.9%) compared to smokers (7.3%). Alcohol consumption is not significantly associated with any form of abuse (Table 5).

**Table 5:** Association of several variables with various forms of abuse [3].

Independent variable		Dependent variable							
		Physical abuse		Emotional/psychological abuse		Financial/material abuse		Neglect/abandonment	
		*f* (percent)	*p* value	*f* (percent)	*p* value	*f* (percent)	*p* value	*f* (%)	*p* value
District	Bhaktapur	21 (15.6)	0.47	20 (14.8)	0.28	20 (14.8)	0.08	13 (9.6)	0.16
	Kathmandu	15 (11.2)		30 (22.4)		18 (13.4)		19 (14.2)	
	Lalitpur	13 (11.2)		22 (19)		27 (23.3)		8 (6.9)	

Age	≤ 68	28 (13.9)	0.54	35 (17.3)	0.51	31 (15.3)	0.41	16 (7.9)	0.13
	> 68	21 (11.5)		37 (20.2)		34 (18.6)		24 (13.1)	

Gender	Male	23 (10.0)	0.06	34 (14.8)	0.02^∗^	33 (14.4)	0.12	24 (10.5)	1
	Female	26 (16.7)		38 (24.4)		32 (20.5)		16 (10.3)	

Ethnicity	Brahmin/Chhetri	29 (14.6)	0.31	34 (17.1)	0.27	33 (16.6)	0.82	21 (10.6)	0.51
	Janajati	19 (10.5)		36 (19.9)		32 (17.7)		18 (9.9)	
	Dalit	1 (20.0)		2 (40.0)		0 (0)		1 (20.0)	

Religion	Hinduism	42 (12.1)	0.31	64 (18.5)	0.82	59 (17.1)	0.83	34 (9.8)	0.41
	Others	7 (17.9)		8 (20.5)		6 (15.4)		6 (15.4)	

Marital status	Married	37 (12.8)	1	48 (16.6)	0.07	51 (17.6)	0.53	29 (10)	0.7
	Others	12 (12.5)		24 (25)		14 (14.6)		11 (11.5)	

Educational status	Illiterate	26 (18.8)	0.01^∗^	35 (25.4)	0.01^∗^	27 (19.6)	0.32	18 (13)	0.22
	Literate	23 (9.3)		37 (15)		38 (15.4)		22 (8.9)	

Employment status	Currently employed	3 (7.0)	0.33	4 (9.3)	0.1	7 (16.3)	1	5 (11.6)	0.79
	Unemployed	46 (13.5)		68 (19.9)		58 (17.0%)		35 (10.2)	

Live with	Alone	1 (4.5)	0.34	1 (4.5)	0.09	2 (9.1)	0.56	3 (13.6)	0.49
	With someone	48 (13.2)		71 (19.6)		63 (17.4)		37 (10.2)	

No. of children not with you	Less than 2	30 (16)	0.03^∗^	42 (22.5)	0.14	41 (21.9)	0.14	17 (9.1)	0.64
	2 and above	6 (6.8)		13 (14.8)		12 (13.6)		6 (6.8)	

No. of children with not you	Less than 3	30 (13.2)	1	47 (20.6)	0.69	39 (17.1)	0.06	17 (7.5)	0.25
	3 and above	6 (12.8)		8 (17)		14 (29.8)		6 (12.8)	

Personal saving	Yes	23 (13.3)	0.87	37 (21.4)	0.23	27 (15.6)	0.58	21 (12.1)	0.31
	No	26 (12.3)		35 (16.5)		38 (17.9)		19 (9.0)	

Alcohol consumption	Yes	26 (10.6)	0.11	41 (16.7)	0.17	41 (16.7)	0.88	22 (8.9)	0.22
	No	23 (16.5)		31 (22.3)		24 (17.3)		18 (12.9)	

Smoking	Yes	27 (9.9)	0.01^∗^	48 (17.6)	0.39	43 (15.8)	0.37	20 (7.3)	0.003^∗^
	No	22 (19.6)		24 (21.4)		22 (19.6)		20 (17.9)	

*Note:* The significance of “^∗^” means significant at 5% level (*p* value < 0.05).

The findings related to the analysis of factors associated with elderly abuse are shown in Table 6. The analysis focuses on the influence of various sociodemographic factors on the likelihood of experiencing different forms of elderly abuse: physical, emotional/psychological, financial/material, and neglect or abandonment. The analysis presents both crude odds ratio (COR) and adjusted odds ratio (AOR) with 95% confidence intervals (CIs). Sexual abuse was excluded from the analysis due to its very low frequency (only three cases), which limits the ability to draw statistically meaningful conclusions.

**Table 6:** Binary logistic regression analysis of factors associated with elder abuse [3].

Independent variable		Dependent variable							
		Physical abuse		Emotional/psychological abuse		Financial/material abuse		Neglect/abandonment	
		COR (95% CI)	AOR (95% CI)	COR (95% CI)	AOR (95% CI)	COR (95% CI)	AOR (95% CI)	COR (95% CI)	AOR (95% CI)
District	Bhaktapur	1.46 (0.69–3.06)	—	0.74 (0.38–1.44)	—	0.57 (0.30–1.08)	—	1.43 (0.57–3.60)	—
	Kathmandu	0.99 (0.45–2.19)	—	1.23 (0.66–2.28)	—	0.51 (0.26–0.98)^∗^	—	2.23 (0.94–5.30)	—
	Lalitpur	1	—	1	—	1	—	1	—

Age	≤ 68	0.82 (0.49–1.38)	—	1.82 (0.16–20.24)	—	0.79 (0.46–1.35)	—	0.57 (0.29–1.11)	—
	> 68	1	—	1	—	1	—	1	—

Gender	Female	1.79 (0.98–3.27)	1.77 (0.76–4.09)	1.84 (1.10–3.09)^∗^	1.53 (0.87–2.68)	1.53 (0.89–2.61)	—	0.97 (0.50–1.90)	—
	Male	1	1	1	1	1	—	1	—

Ethnicity	Brahmin/Chhetri	1.45 (0.78–2.69)	—	0.83 (0.49–1.39)	—	0.92 (0.54–1.57)	—	1.06 (0.55–2.07)	—
	Dalit	2.13 (0.22–20.06)	—	2.68 (0.43–16.67)	—	NA	—	2.26 (0.24–21.36)	—
	Janajati	1	—	1	—	1	—	1	—

Religion	Hinduism	0.63 (0.26–1.52)	—	0.88 (0.39–2.01)	—	1.13 (0.45–2.82)	—	0.59 (0.23–1.53)	—
	Others	1	—	1	—	1	—	1	—

Marital status	Married	1.03 (0.51–2.06)	—	0.59 (0.34–1.04)	—	1.25 (0.66–2.38)	—	0.86 (0.41–1.79)	—
	Others	1	—	1	—	1	—	1	—

Educational status	Illiterate	2.26 (1.23–4.14)^∗^	1.93 (0.85–4.40)	1.93 (1.14–3.24)^∗^	1.63 (0.92–2.86)	1.33 (0.77–2.30)	—	1.53 (0.79–2.97)	—
	Literate	1	—	1	1	1	—	1	—

Employment status	Currently employed	0.48 (0.14–1.62)	—	0.41 (0.14–1.19)	—	0.95 (0.40–2.24)	—	1.15 (0.43–3.12)	—
	Unemployed	1	—	1	—	1	—	1	—

No. of children with you	Less than 2	0.38 (0.15–0.95)^∗^	—	0.59 (0.30–1.18)	—	0.56 (0.27–1.13)	—	0.73 (0.27–1.92)	—
	2 and above	1	—	1	—	1	—	1	—

No. of children with not you	Less than 3	0.96 (0.37–2.47)	0.70 (0.16–1.88)	0.79 (0.34–1.80)	—	2.05 (1.01–4.19)^∗^	—	1.81 (0.67–4.88)	—
	3 and above	1	—	1	—	1	—	1	—

Personal savings	No	1.09 (0.60–2.0)	—	1.37 (0.82–2.29)	—	0.84 (0.49–1.45)	—	1.40 (0.72–2.70)	—
	Yes	1	—	1	—	1	—	1	—

Alcohol consumption	No	0.59 (0.32–1.09)	—	0.69 (0.41–1.17)	—	0.95 (0.55–1.66)	—	0.66 (0.34–1.27)	—
	Yes	1	—	1	—	1	—	1	—

Smoking	No	0.44 (0.24–0.82)^∗^	0.38 (0.17–0.81)	0.78 (0.45–1.35)	—	0.76 (0.43–1.35)	—	0.36 (0.18–0.70)^∗^	—
	Yes	1	1	1	—	1	—	1	—

*Note:* The significance of “^∗^” means significant at 5% level (*p* value < 0.05).

Compared to Lalitpur, the odds of experiencing physical abuse in Bhaktapur (AOR: 1.20; 95% CI: 0.48–2.99) and Kathmandu (AOR: 0.56; 95% CI: 0.19–1.61) are not statistically significant. The adjusted odds of emotional abuse are lower in Bhaktapur (AOR: 0.59; 95% CI: 0.27–1.30) and Kathmandu (AOR: 0.81; 95% CI: 0.37–1.77) compared to Lalitpur, though these differences are not significant. The odds are significantly lower in Kathmandu (AOR: 0.32; 95% CI: 0.13–0.76) compared to Lalitpur, indicating a reduced risk of financial abuse in Kathmandu. No significant differences are observed across districts.

Elderly individuals aged ≤ 68 years have higher odds of physical abuse (AOR: 1.26; 95% CI: 0.52–3.04) than those > 68, but this difference is not significant. No significant difference is observed based on age. Elderly individuals aged ≤ 68 years have significantly lower odds of neglect (AOR: 0.22; 95% CI: 0.07–0.71) compared to those > 68 years.

Females have significantly higher odds of emotional abuse (COR: 1.84; 95% CI: 1.10–3.09), although the adjusted odds (AOR: 1.44; 95% CI: 0.68–3.08) become nonsignificant. Although females have higher odds, the associations are not statistically significant after adjustment.

Brahmin/Chhetri elderly individuals have significantly higher odds of neglect (AOR: 4.45; 95% CI: 1.29–15.24) compared to Janajati. This highlights an elevated risk of neglect among Brahmin/Chhetri. The differences across ethnic groups are not statistically significant.

Married individuals have significantly higher odds of neglect (AOR: 4.18; 95% CI: 1.05–16.70) compared to unmarried, widowed, or separated individuals. Married individuals have lower odds of emotional abuse, but the results are not statistically significant.

Illiterate elderly individuals have higher odds of physical (COR: 2.26; 95% CI: 1.23–4.14) and emotional abuse (COR: 1.93; 95% CI: 1.14–3.24) compared to literate individuals, although the adjusted odds are not significant. No significant differences are observed based on education.

Elderly individuals with fewer than three children not living with them have significantly higher odds of financial abuse (COR: 2.05; 95% CI: 1.01–4.19), though the adjusted odds become nonsignificant.

Nondrinkers have significantly lower odds of physical abuse (AOR: 0.33; 95% CI: 0.12–0.87) compared to drinkers. Nonsmokers have significantly lower odds of physical abuse (AOR: 0.41; 95% CI: 0.17–0.97) and neglect (AOR: 0.26; 95% CI: 0.09–0.79) compared to smokers.

## 4. Discussion

This study sheds light on the critical issue of elderly abuse and the health challenges faced by the aging population in the Kathmandu Valley. Through its findings, this report underscores the importance of recognizing and addressing the multifaceted vulnerabilities that elderly individuals face, particularly concerning abuse, chronic health conditions, and the impact of sociodemographic factors. In our study, 12.7% of elderly individuals reported experiencing physical abuse, with serious instances such as being beaten or gripped forcefully. This prevalence is substantially lower than the 49.1% reported in a study [16], which included broader definitions of abuse and possibly larger sample sizes and geographic variations. The lower prevalence observed here could suggest underreporting due to societal stigmas or fear of retaliation. It also indicates a potential need for interventions aimed at increasing awareness and providing safe spaces for elderly individuals to report abuse.

Emotional abuse was the most common type of abuse reported in our study, affecting 18.7% of participants, likely due to psychological stressors inflicted by family members or caregivers. This rate is lower than the 34.9% reported by Baral et al. [3] and the 20.6% psychosocial abuse noted by Acharya et al. [1]. Our findings indicate that although neglect is prevalent, it appears less frequently in Kathmandu Valley, potentially due to strong familial bonds or community support. However, the cases reported underline the necessity of ensuring elderly care, especially during health crises, and highlight the potential role of community programs in supporting caregivers. Information on perpetrators of abuse was not systematically collected in this study, which is a key limitation. Evidence from South Asian studies indicates that most elder mistreatment occurs within family settings—most often by adult children, spouses, or close relatives. Including perpetrator data in future research would help identify high‐risk relationship dynamics and guide targeted family‐based interventions.

Sexual abuse was extremely rare in this study, affecting only 0.8% of participants. Other studies, such as Yadav and Paudel, report higher prevalence rates (3.3%), suggesting that sexual abuse may be underreported in our sample due to cultural taboos and stigma around discussing sexual violence. Although rare, the existence of sexual abuse cases underscores the need for comprehensive elder protection laws and the establishment of safe channels for reporting abuse without stigma. Female participants were more likely to experience emotional abuse in our study, which aligns with Baral et al.’s finding that women are at higher risk of abuse. This gender disparity can be interpreted within Nepal’s sociocultural context, where patriarchal family structures and economic dependency place elderly women—particularly widows—at higher risk of emotional neglect and mistreatment. Similar patterns have been documented in India and Malaysia, where social isolation, loss of financial control, and widowhood increase vulnerability to abuse. These findings highlight the need for gender‐sensitive elder protection and social support programs. This disparity likely reflects the traditional gender roles and social structures that place elderly women in more vulnerable positions within households. Brahmin/Chhetri individuals were at a higher risk of neglect, a finding that points to cultural and familial expectations that may place strain on family caregivers, leading to neglect. This is consistent with findings from another study [17], which noted similar vulnerabilities associated with ethnic and social factors. Elderly individuals over 68 years old had higher odds of neglect, a trend that may reflect physical or cognitive declines associated with aging, increasing dependency on caregivers. Illiteracy was associated with increased odds of both physical and emotional abuse, which may be due to reduced awareness of rights and lower social status. This highlights the importance of promoting educational programs targeting elder rights and protections. Both smoking and alcohol consumption were associated with higher risks of physical abuse and neglect, echoing findings from another study [17] that linked substance use with higher rates of mistreatment. Substance abuse interventions and support could potentially reduce the incidence of abuse among elderly individuals who struggle with addiction. Hypertension (21.4%) and diabetes (13.7%) were common self‐reported health issues in our study. This aligns with findings from the UN DESA Population Division [18], which reported a high prevalence of circulatory and other health issues, such as musculoskeletal and digestive ailments. Additionally, Kshetri et al. noted a higher prevalence of hypertension (37.85%) in urban elderly populations [6]. The high rates of chronic illnesses suggest that elderly individuals in Kathmandu Valley may benefit from improved access to regular medical checkups and specialized geriatric care. Health programs tailored to address hypertension, diabetes, and musculoskeletal health could be especially beneficial. This study underscores the multifaceted nature of elder abuse in Kathmandu Valley and its association with various sociodemographic and health factors. Despite lower abuse rates compared to other studies, significant proportions of elderly individuals experience emotional, financial, and physical abuse. In the neighboring country such as India, 80% of older adults have at least one chronic health problem followed by 77% who have at least two chronic health problems [19]. However, hypertension was one of the chronic health problems faced in India, which was treatable [11]. The study conducted in Iran shows that 40% of participants underwent any one type of abuse in which financial abuse was 32%. The study also shows that there is a statistically significant relationship between some demographic values such as age, income, and elder abuse. The findings of the results highlight the need to develop elder‐related policies in the country.

The study’s cross‐sectional design limits causal inference, and data on perpetrators were not collected. The predominance of urban, Hindu, and Brahmin/Chhetri participants may limit generalizability. Future studies should include diverse geographic and ethnic groups, integrate qualitative exploration of family dynamics, and adopt standardized WHO instruments for cross‐national comparability.

## 5. Conclusions

This study examines the prevalence, types, and factors associated with elder abuse in Kathmandu Valley. Emotional abuse was the most common, followed by financial abuse, neglect, and physical abuse, whereas sexual abuse was rarely reported, likely due to stigma. Economic dependency, health vulnerabilities, and family dynamics contribute to mistreatment. Chronic health conditions, including hypertension, diabetes, and musculoskeletal disorders, increase elderly dependence on caregivers, heightening the risk of neglect. Factors such as gender, age, ethnicity, education, marital status, and substance use significantly influence the likelihood of abuse, reflecting the sociocultural complexities of elder care.

## Conflicts of Interest

The authors declare no conflicts of interest.

## Funding

This study was funded by the University Grants Commission‐Nepal.

## Data Availability

The data that support the findings of this study are available from the corresponding author upon reasonable request.
